# Allosteric regulation of histone lysine methyltransferases: from context-specific regulation to selective drugs

**DOI:** 10.1042/BST20200238

**Published:** 2021-03-26

**Authors:** Chen Davidovich, Qi Zhang

**Affiliations:** 1 Department of Biochemistry and Molecular Biology, Biomedicine Discovery Institute, Faculty of Medicine, Nursing and Health Sciences, Monash University, Clayton, Victoria, Australia; 2 EMBL-Australia and the ARC Centre of Excellence in Advanced Molecular Imaging, Clayton, Victoria, Australia

**Keywords:** allosteric regulation, histone lysine methyltransferases, set domain

## Abstract

Histone lysine methyltransferases (HKMTs) are key regulators of many cellular processes. By definition, HKMTs catalyse the methylation of lysine residues in histone proteins. The enzymatic activities of HKMTs are under precise control, with their allosteric regulation emerging as a prevalent paradigm. We review the molecular mechanisms of allosteric regulation of HKMTs using well-studied histone H3 (K4, K9, K27 and K36) methyltransferases as examples. We discuss the current advances and future potential in targeting allosteric sites of HKMTs for drug development.

## Introduction

**Figure 1.**
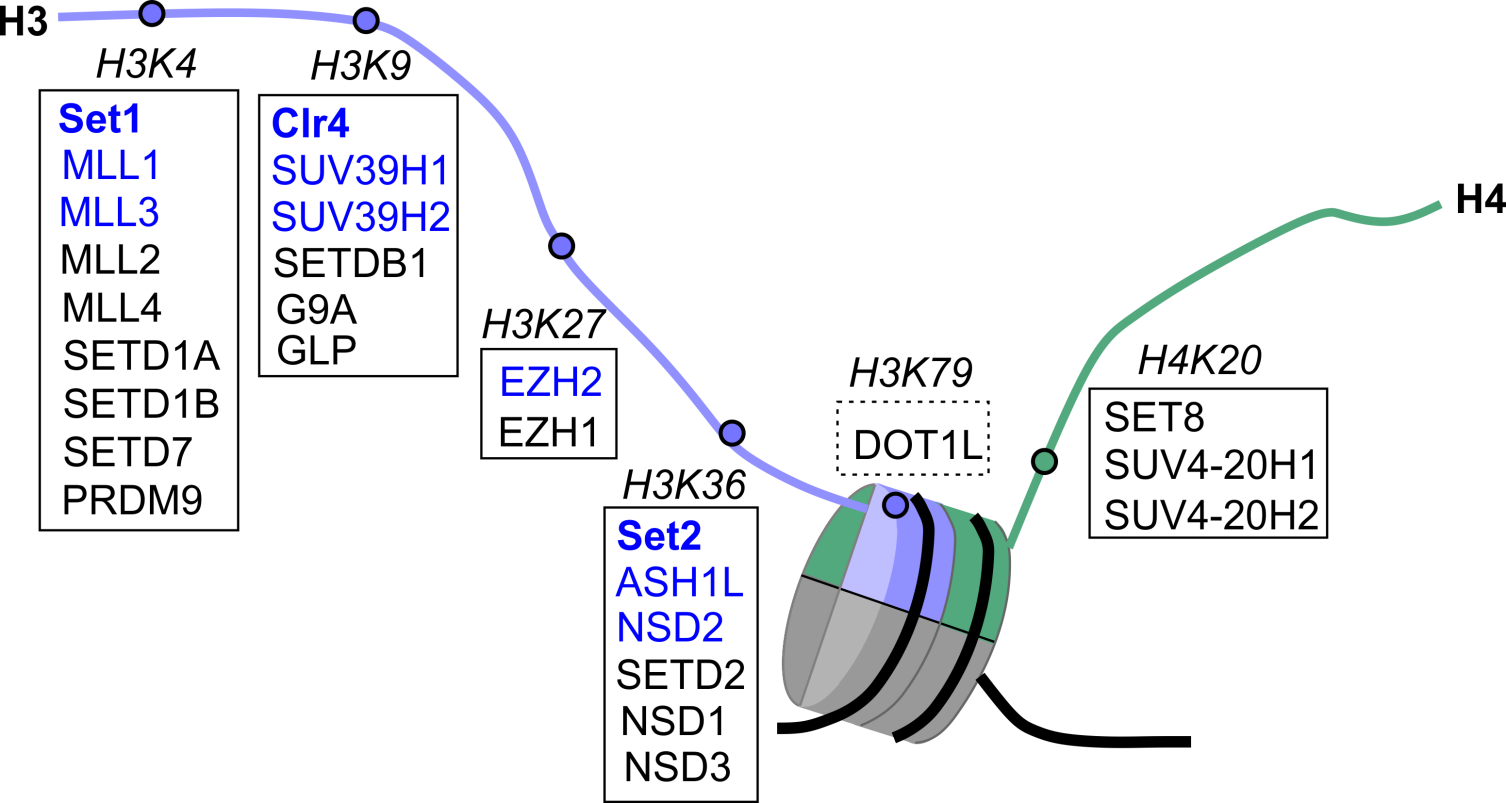
Schematic presentation of histone lysine methylation marks on histones H3 and H4 with their corresponding HKMTs. Methyltransferases for each of the lysine substrates are shown in boxes. Boxes of continuous lines include SET domain HKMTs. Fungal HKMTs are represented in bold font and human HKMTs are in regular font. In blue, are HKMTs that undergo allosteric regulation and are discussed herein.

Histones are subject to covalent post-translational modifications, such as lysine methylation [1–3]. Lysine methylation occurs on multiple lysine residues of histones, and exists in three methylation states: mono-, di- and tri-methylation, referred to as me1, me2 and me3, respectively. The various lysines and their different methylation levels greatly increases the complexity of the information they deliver [4,5]. Accordingly, these lysine methylations are linked to a wide range of chromatin-related processes. These include transcriptional regulation, nuclear organisation, replication and DNA repair [6,7]. Histone lysine methyltransferases (HKMTs) transfer a methyl group from the methyl donor S-adenosyl-l-methionine (SAM) to the ε-amino group of a selected lysine residue [4,8]. Target lysines are mainly found in histones H3 and H4 and define different groups of HKMTs, clustered based on the specific lysine they target [4,8] (Figure 1). Most HKMTs contain an evolutionarily conserved catalytic SET domain, standing for Su(var)3–9, Enhancer of Zeste and Trithorax [9]. An exception is the histone H3 lysine 79 (H3K79) methyltransferase DOT1L, which is not covered in this review [10].

As with other enzymes involved in key biological and cellular processes, the activity of HKMTs is tightly controlled by various mechanisms [4,11]. This review focuses on the allosteric regulation of HKMTs, which allows them to sense their environment and have their catalytic activity adjusted accordingly.

Allostery is considered an inherent property of many enzymes [12]. In this review, we adhere to the definition of allosteric regulation of an enzyme as previously established [13–16]: the stabilisation of an activated conformation of an enzyme upon its binding to an allosteric effector. The allosteric effector could be another protein or a ligand that can reversibly bind to the enzyme. The interactions between the effector and the enzyme lead to an ‘allosteric event’, which stabilises the enzyme in an active conformation to increase its catalytic efficiency. Hence, effectors such as modified histone tails, cell type-specific protein subunits, or DNA, allows for the allosteric regulation of HKMTs in a context-specific manner, with specific examples discussed below.

The dysregulation of HKMTs is commonly linked to human diseases, making them attractive therapeutic targets [11,17,18]. However, the development of selective drugs to target the catalytic site of specific HKMTs remains challenging. A contributing factor is the structural resemblance between active sites of different HKMTs, especially for the structurally conserved SET domain proteins. Understanding how allosteric regulation of HKMTs takes place will open paths for the development of new selective drugs [19–21].

## Flexible regions near the SET domain provide means for the allosteric regulation of HKMTs

The major type of histone lysine methyltransferases is SET domain-containing enzymes. The SET domain is relatively small, with ∼130–140 amino acids. SET domains are commonly associated with other domains to form a multi-domain protein [9]. The mobile C-terminal region of the SET domain — termed post-SET — forms the substrate binding channel together with the SET-I (SET insert) domain [9,22]. The post-SET is flexible in nature, which is important for the substrate turnover [23] and allows it to participate in the allosteric regulation of catalysis. A conformational change or rearrangement of the post-SET loop is often required for the full enzymatic activity and the product specificity of SET domain proteins [19].

In some multi-subunit enzymatic complexes, the conformational change of the post-SET loop is facilitated by other subunits in the same complex. There are two common mechanisms for subunit-induced allosteric regulation: (1) another subunit in the same enzymatic complex can act as an allosteric effector or (2) can host the allosteric regulatory site that binds another effector. These two mechanisms are not mutually exclusive and can appear simultaneously or intermittently in the same complex or in closely related complexes. In this review, we discuss how HKMTs are allosterically regulated. In particular, we discuss four distinct types of SET domain-containing HKMTs with clear evidence to support their allosteric regulation: EZH2, Clr4/SUV39, a few members of H3K36 methyltransferases, and the MLL family.

## Allosteric regulation of the H3K27 methyltransferase PRC2 by its subunits and histone effectors

Polycomb repressive complexes (PRCs) are histone modifier complexes formed by members of the polycomb-group (PcG) protein family [24]. The polycomb repressive complex 2 (PRC2) is essential for embryonic development and for the maintenance of cell identity. At the molecular level, PRC2 maintains the repressed state of developmentally expressed genes in multicellular organisms [25–27]. At the biochemical level, PRC2 marks genes for repression by the tri-methylation of lysine 27 on histone H3 (H3K27me3), a hallmark of facultative heterochromatin [25–27]. The dysregulation of PRC2 is frequently linked to human disease, such as cancer and congenital disorders [28,29].

**Figure 2.**
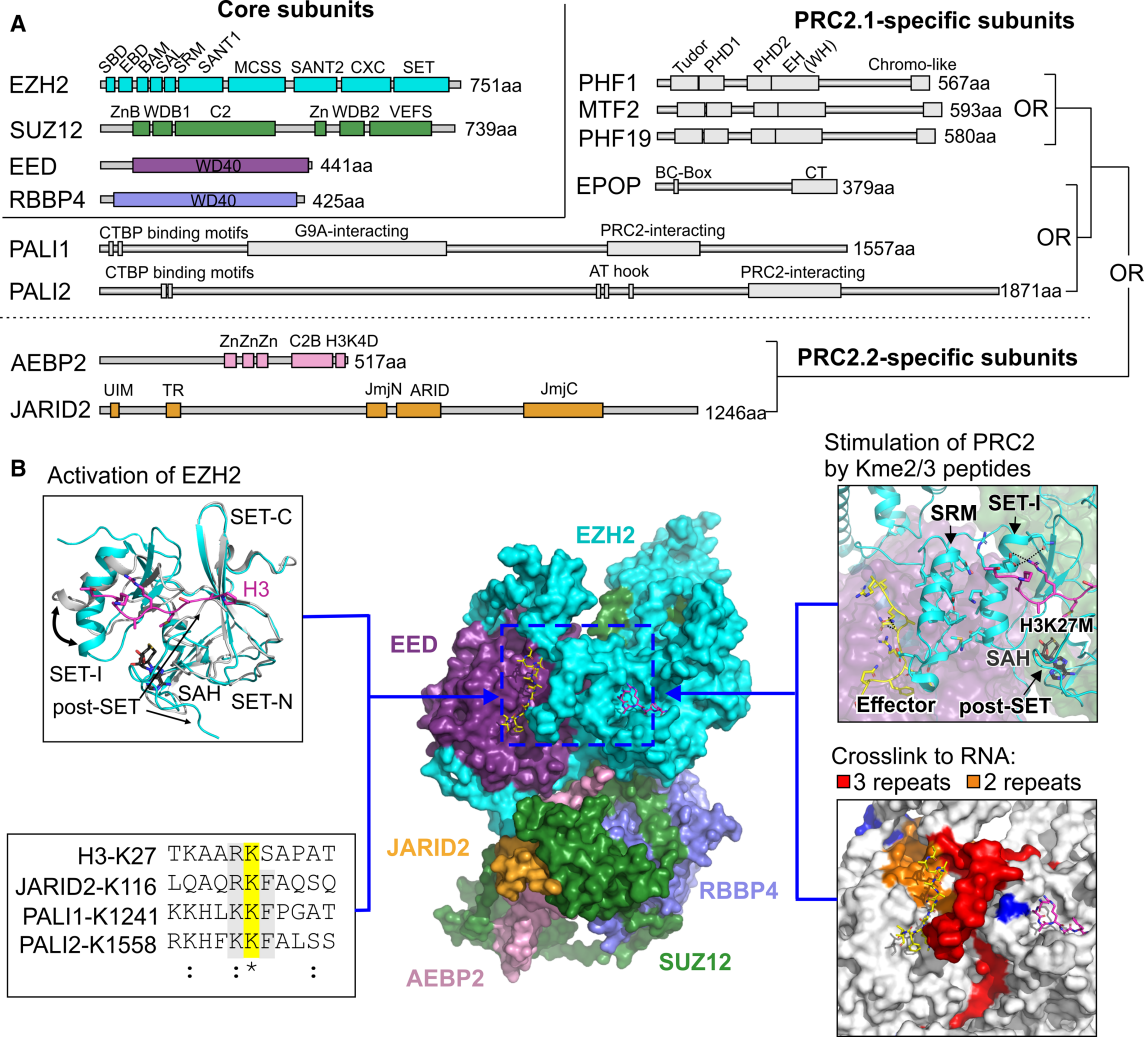
The allosteric regulation of PRC2. (**A**) Domain organisation of the PRC2 core subunits (top left) and accessory subunits of the PRC2.1 and PRC2.2 complexes, as indicated. (**B**) Structural basis for the allosteric regulation of PRC2. Middle panel: Cryo-EM structure of PRC2.2 (PDB: 6C23 [46]). Subunits colours used as in panel (**A**). Top left panel: when EZH2 is bound to EED and SUZ12, the SET-I and post-SET motifs undergo a conformational change (indicated in black arrows). In grey: the structure of the SET domain from EZH2 in isolation (PDB: 4MI5 [36]); in cyan: the SET domain from the structure of EZH2 in a complex with EED and SUZ12 (PDB: 5HYN [40]), including the SAH (in CPK colouring) and an H3K27M peptide (labelled H3, in magenta) in the substrate channel. Top right panel: stimulation of PRC2 by methylated lysine peptides through interactions between the di/tri-methyl-lysine effector, the SRM and the SET-I regions (PDB: 6C23 [46]). The allosteric effector (JARID2-K116me3) is in yellow and the H3K27M peptide in the substrate channel is in magenta. Bottom left: multiple sequence alignment of different allosteric effectors of PRC2. The substrate lysine is highlighted in yellow. The conserved lysine or arginine at the −1 position and the phenylalanine in the +1 position are in grey, with the latter conserved between JARID2, PALI1 and PALI2, but not the canonical substrate H3. Bottom right: The RNA-binding sites on the regulatory center of PRC2 are highlighted in red, orange or blue if detected in three, two or one independent RBDmap experiments [55], respectively.

The core PRC2 complex comprises four subunits: one of the catalytic subunits EZH2 or EZH1, the WD40-containing regulatory protein EED, one of the two WD40 proteins RBBP4 or RBBP7, and the scaffold protein SUZ12 [30] (Figure 2A). In addition, multiple accessory subunits associate with the PRC2 core complex to modulate its recruitment to chromatin and its enzymatic activity [30]. In metazoans, the accessory subunits define two types of holo-PRC2 complexes: PRC2.1 and PRC2.2 (Figure 2A) [30]. In vertebrates, the PRC2.1 contains one of the polycomb-like proteins — PHF1, MTF2 or PHF19 — and can include one additional accessory subunit: either EPOP, PALI1 or PALI2 [31,32]. The PRC2.2 complex contains the accessory subunits JARID2 and AEBP2, which are conserved from fly to human [33,34].

The isolated SET domain of EZH2 is in an autoinhibited state [35–37]. The SET-I and post-SET motifs adopt a conformation which blocks the histone tail binding and forms an incomplete binding pocket for SAM (Figure 2B, top left, structure in grey colour) [35–37]. When EZH2 is in a complex with EED and SUZ12, the SET domain within the ternary complex can undergo a structural rearrangement between autoinhibited, basal, and stimulated states [38]. In its basal or stimulated states, the autoinhibition state of EZH2 is alleviated: the histone substrate-binding site is exposed and the SAM substrate-binding site is formed (Figure 2B, top left, structure in cyan colour) [38]. The enzymatic activity of EZH2 can be further stimulated by its own product: the H3K27me3 mark. This has been referred to as a ‘write and read' mechanism [28].

Mechanistically, the regulatory subunit EED is the ‘reader', which includes a binding site for the H3K27me3 effector. Upon binding to EED, the H3K27me3 effector triggers allosteric activation of EZH2, which serves as the ‘writer'. The mechanism for H3K27me3-induced allosteric activation of PRC2 has been identified by multiple independent structural and functional works [38–41]. These studies revealed that the H3K27me3 mark binds to PRC2 [42] through an aromatic cage in EED, located on the top of the β-propeller WD40 structure [39,43]. H3K27me3-binding leads to the rearrangement of the stimulatory-responsive motif (SRM) within EZH2, which is arranged from a disordered conformation to an α-helix structure [38,40]. This rearrangement of the SRM forms extensive hydrophobic interactions between the SRM and the nearby SET-I, which in turn stabilises the conformation of the active site. These conformational rearrangements shape the catalytic center of EZH2 and promote the enzymatic activity of PRC2 [38]. Indeed, disruption of the interactions between the SRM to the SET-I, using α-helical mimetics, selectively inhibits the allosteric activation of PRC2 [44]. Similarly, point mutations at the interfaces between the SRM and either the SET-I domain or EED abolish the stimulation of H3K27me3 *in vitro* and reduce the global H3K27me2/3 levels *in vivo* [44].

H3K27me2/3 is not the only effector of PRC2: PRC2 can also be subjected to the allosteric stimulation by the di- or tri-methyl lysine 116 of the PRC2.2-specific subunit JARID2 (JARID2-K116me2/3) [45]. JARID2-K116 can serve as a non-histone substrate for PRC2, where the JARID2-K116me2/3 product can then stimulate PRC2. The mechanism for JARID2-K116me2/3-induced stimulation of PRC2 resembles that of H3K27me3: JARID2-K116me2/3 binds to the aromatic cage of EED and stabilises the SRM to stimulate PRC2 (Figure 2B, top right) [40,45,46]. Through allosterically activating PRC2, JARID2 is proposed to facilitate the deposition of H3K27me3 *de novo*, without a dependency on a pre-existing H3K27me3 mark for allosteric activation [45]. By doing so, JARID2 K116me2/3 is proposed to ‘jump start' H3K27me3 deposition, which can later facilitate its own maintenance through H3K27me3-induced allosteric activation of PRC2 [28].

The physiological relevance of JARID2-induced allosteric activation of PRC2 in the context of embryonic development is yet to be determined. However, some indication that this molecular mechanism is fundamental can be obtained from evolutionary perspectives. First, JARID2 K116 is conserved from fly to human [45]. Second, we recently found that the vertebrate-specific subunits of PRC2.1, PALI1 and PALI2, adopt the same mechanism as JARID2 to allosterically activate PRC2 [47] (Figure 2B, bottom left). Mechanistically, PRC2 di- and tri-methylates PALI1 K1241 (PALI1 K1241me2/3) and possibly PALI2 K1558, which can in turn allosterically activate PRC2 [47]. Despite this mechanistic resemblance, PALI1 and PALI2 do not share a common ancestor with JARID2, with the latter conserved from fly to human and the formers are vertebrate specific [47]. This implies that PALI1/2-induced allosteric activation of PRC2 emerged through a convergent evolution with JARID2 [47]. More broadly, it suggests that subunit-induced allosteric activation of PRC2 is an indispensable molecular property of holo-PRC2 complexes in vertebrates [47].

RNA also plays a role in the regulation of PRC2. PRC2 binds to RNA promiscuously [48–50], with a preference for prevalent sequences of consecutive guanines [51]. RNA is proposed to regulate PRC2 in various mechanisms, including the recruitment of PRC2 to its target genes, the eviction of PRC2 from chromatin, and the inhibition of PRC2 by either preventing its HMTase activity or by competing with DNA for binding sites, to name a few (reviewed in [52–54]). We and others have identified several RNA binding sites within PRC2 complexes [46,55,56]. We found that one of the RNA-binding patches in PRC2 largely overlaps with the H3K27me3 binding site in EED and the SRM in EZH2 (Figure 2B, bottom right, with the RNA-binding sites highlighted in red, orange and blue) [55]. Accordingly, we observed that either an H3K27me3 peptide or a JARID2-K116me3 peptide alleviates the RNA-mediated inhibition of PRC2 to some extent [55]. It is unknown if RNA directly affects the allosteric activation of PRC2, but the overlap between the RNA-binding site to the regulatory center of PRC2 provides means for that.

‘Crosstalk' between histone modifications is common in the regulation of histone methyltransferases. It occurs when a histone mark is deposited by one histone modifier and then regulates another modifier. PRC2 is regulated by various histone marks. For instance, H3K36me2/3 or H3K4me3 inhibit the HMTase activity of PRC2 [57]. A mechanism for an allosteric inhibition has been proposed, based on the observation that the H3K4me3 modification directly affects the catalytic efficiency but not the affinity to the substrate [57]. More recently, a cryo-EM structure of PRC2 with a di-nucleosomal construct, complemented by functional assays, led to propose a molecular mechanism for the inhibition of PRC2 by the H3K36 methyl mark [58]: in the substrate nucleosome, the unmodified H3K36 site is sandwiched at the interface between EZH2 and the nucleosomal DNA [58]. According to the proposed mechanism, the correct positioning of the nucleosome substrate is allowed only when H3K36 is unmethylated, and this enables the correct presentation of the H3 tail to the catalytic centre in EZH2 [58]. While this mechanism does not fit with the definition of allosteric regulation followed in this review (see above), it does fit with a broader definition sometimes used, referred to the interaction of an effector with a site other than the active site of the enzyme. Consistent with this mechanism, a recent cryo-EM study of PRC2–AEBP2–JARID2 in a complex with H3K4me3-containing nucleosome revealed two distinct states of the H3 tail [59]. Importantly, only in one of these conformations, the H3 tail is engaged with the SET domain of EZH2 [59]. The presence of such two conformations of the H3 tail, where only one of them allows catalysis, could explain why H3K4me3-modified nucleosome is a sub-optimal substrate comparing an unmodified nucleosome [59], despite both having a similar affinity to PRC2 [57]. Collectively, these recent structures imply that the H3K4me3 [59] and H3K36me3 [58] marks reduce the activity of PRC2 by inducing a suboptimal presentation of the H3 tail to the active site. Future kinetic assays with mutagenesis, guided by these recent structures, will likely allow deciphering these molecular mechanisms and to directly support them.

Overall, the mechanism of allosteric regulation is well established in PRC2: once the subunits EED and SUZ12 associate with EZH2, the autoinhibition state is released. Following that release, PRC2 is in its basal state, with the complete substrate-binding site being formed. From that point, the SET domain of EZH2 can participate in methyl transfer. Yet, in its basal state, EZH2 has a relatively low catalytic efficiency. Once the H3K27me2/3 product is produced it is recognised by the regulatory subunit EED, which then triggers a series of conformational changes in EZH2 to stabilise its stimulated state. PRC2 can be subjected to allosteric activation even in the absence of the H3K27me3 mark: subunit-induced allosteric activation of PRC2 occurs when a di- or tri-methyl-lysine within the accessory subunit PALI1/2 or JARID2 leads to the allosteric activation of PRC2.1 or PRC2.2, respectively. The various effectors and mechanisms for the allosteric regulation of PRC2 enable its regulation in a context-specific manner and open paths for drug development (more below).

## Allosteric regulation of the H3K9 methyltransferase SUV39H1 facilitates a read-write mechanism

**Figure 3.**
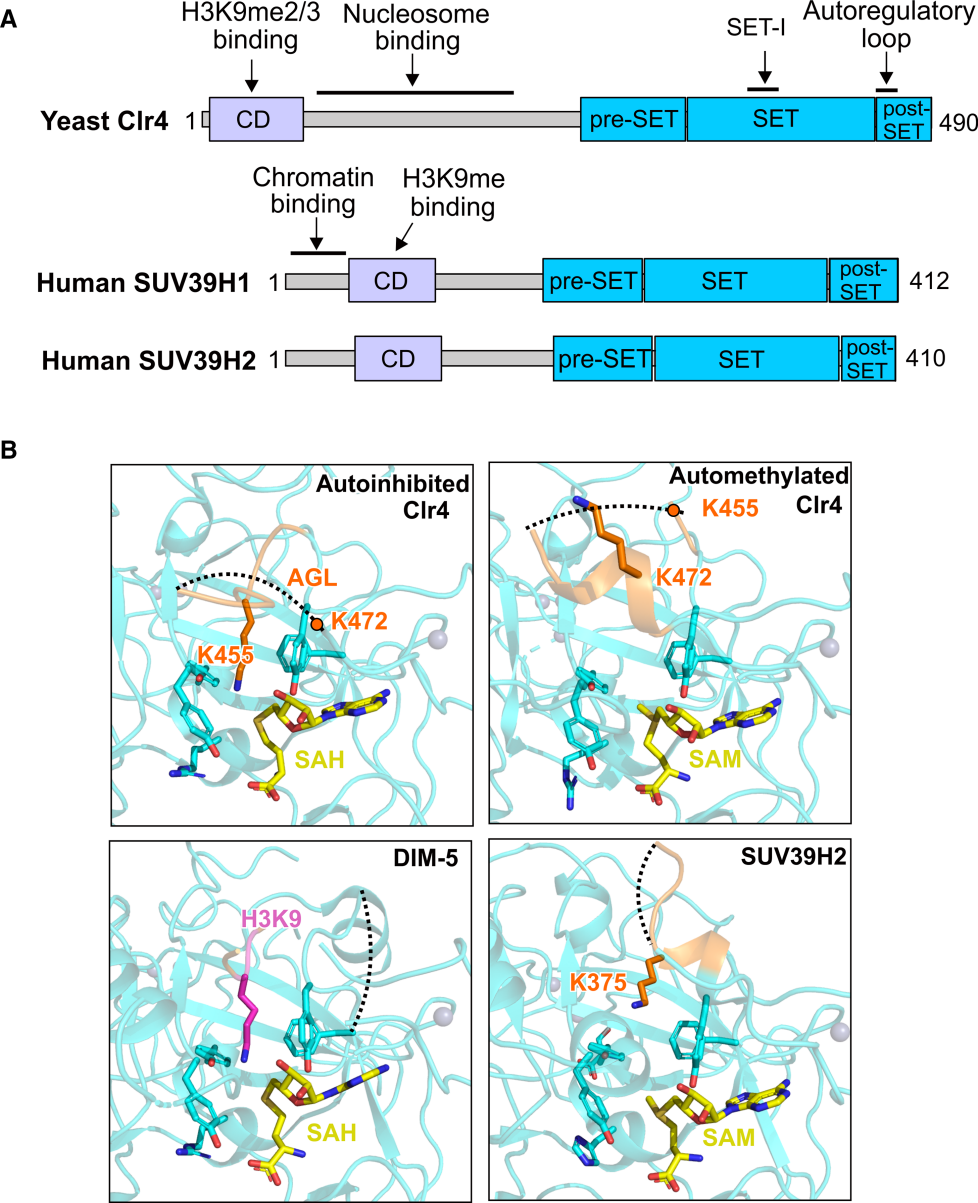
An autoregulatory loop shapes the active site of the H3K9 methyltransferases Clr4 and SUV39H1/2. (**A**) Domain organisation of yeast Clr4 (top) and human SUV39H family (bottom). (**B**) Clr4 K455 (top left, PDB: 6BOX [68]) and human SUV39H2 K375 (bottom right, PDB: 2R3A [22]) are located in the substrate binding pocket, in a similar position to H3K9 substrate bound to DIM-5 (bottom left, PDB: 1PEG [69]). The automethylation of Clr4 K455 (top right, PDB: 6BP4 [68]) results in a conformational change of the autoregulatory loop that makes the substrate binding-site accessible. In all boxes, the autoregulatory loop regions are in orange, SAH or SAM are in yellow sticks and H3 substrate is in magenta. Black dashed lines represent disordered regions.

The di- and tri- methylation of H3K9 (H3K9me2 and H3K9me3, respectively) are a hallmark of constitutive heterochromatin. H3K9me2/3 serves as a platform to recruit heterochromatin protein 1 (HP1) family proteins to mediate gene silencing [60,61]. In fission yeast, Clr4 is the sole H3K9 methyltransferase. In humans, H3K9me2/3 is deposited partially by the Clr4 orthologues SUV39H1/KMT1A and SUV39H2/KMT1B, among other methyltransferases [62–65]. Loss of SUV39H1/2 results in genome instability and impairs heterochromatin formation in mammals [66]. Clr4 and SUV39H belong to the SUV39 sub-family and they share a similar domain architecture: they have a conserved chromodomain (CD) in the N-terminal and a catalytic SET domain in the C-terminal (Figure 3A) [67].

Crystal structures of catalytic domains of Clr4 and SUV39H2 revealed auto-inhibitory conformations [22,68]. A proposed autoregulatory loop (ARL) located between the SET-I and the post-SET, and was shown to block the substrate-binding site in both Clr4 and SUV39H2 [22,68]. Mechanistically, K455 of the Clr4 ARL loop fully inserts into the catalytic pocket [68] (Figure 3B, top left). In comparison, in the active conformation of the H3K9 methyltransferase DIM-5 from *N. crassa*, the catalytic site is occupied by the K9 substrate lysine (Figure 3B, bottom left) [69]. *In vitro* methyltransferase assays and LC–MS/MS confirmed that K455 in Clr4 is automethylated [68]. Mutagenesis suggested another potential methylated residue in the ARL is K472 [68]. As a consequence of these automethylations, the ARL undergoes a conformational change to no longer block the active site (Figure 3B, top right) [68]. In line with these observations, inducing automethylation of Clr4 by pre-incubation with SAM stimulates its activity on histone tail peptide substrates [68]. Mutations that potentially disrupt the autoregulation of Clr4 led to severe defects in yeast [68], implying its functional importance *in vivo*.

Despite no direct evidence yet, automethylation may play a role in the regulation of the human SUV39H1 and SUV39H2. According to the crystal structure of human SUV39H2, K375 (corresponding to Clr4 K455) is inserted halfway into the active site and blocking it [22] (Figure 3B, bottom right). Another residue in the C-terminal of the putative ARL of SUV39H2, K392, has been shown to be automethylated [70]. This residue is also conserved in SUV39H1 and is likely the counterpart of Clr4 K472.

The mechanisms described above for the regulation of H3K9 methyltransferase through automethylation of residues in their ARL does not fit with the definition of allosteric regulation, as defined herein and elsewhere [13–16]. Yet, it does demonstrate the potential of the ARL as a regulatory switch that is evolutionarily conserved from the yeast Clr4 to the human SUV39H1 and SUV39H2. This indicates the requirement for tight regulation of H3K9 methylation by these enzymes and opens the possibility for allosteric regulation by external effectors.

A potential effector of SUV39H family proteins is their own H3K9 methyl mark. The CD domain of both yeast Clr4 and human SUV39H binds to their product: H3K9me2/3 [71]. Accordingly, the CD domain and the H3K9me3 binding site in it are required for spreading of the H3K9me2/3 mark and maintaining heterochromatin domains *in vivo* and *in vitro* [71–73]. A similar read-write positive feedback loop was also shown in the H3K27 methyltransferase PRC2, as described above [38–41]. Collectively, this suggests that read-write feedback loops may be a general property of histone methyltransferases that deposit repressive marks.

Importantly, the recognition of the H3K9me2/3 marks via the CD domain of SUV39H family proteins stimulates catalysis directly, rather than facilitating substrate binding [73,74]. However, the molecular mechanism of how H3K9me2/3 marks promote the catalytic activity of SUV39H family proteins is not fully understood. In the case of the yeast Clr4, experiments using di-nucleosome substrates, with one of the nucleosomes pre-methylated at H3K9, confirmed regulation *in cis* [73]. Similar experiments using mononucleosomes did not reveal stimulation *in trans* [73]. This data led to propose a ‘guided-state’ model. According to the guided-state model, the CD–H3K9me3 interactions guide the SET domain of Clr4 to adopt correct orientation of the H3 tail substrate after the initial substrate binding [73]. A different mechanism has been observed for human SUV39H1, where its HMTase activity is stimulated *in trans* by H3K9me3 peptides. This activity applies only to chromatin substrates, not peptide substrates [75]. A mechanism has been proposed for this stimulation of SUV39H1: first, CD-dependent recognition of nucleosomal H3K9me3 takes place. Upon binding to chromatin, inhibitory interactions between the N-terminal domain of SUV39H1 to the SET domain are released, which allosterically activates the enzyme [75].

The regulatory mechanisms of the yeast and human Clr4/SUV39H HKMTs are not well characterised at the molecular level. Nevertheless, there is ample indirect and some direct evidence for their allosteric regulation. Future structural works would likely determine how the CD domain in Clr4 and SUV39H HKMTs stimulates H3K9 methyltransferase.

## The auto-inhibitory state of H3K36 methyltransferases is relieved by their own subunits and possibly chromatin

Methylation on lysine 36 of H3 is mostly associated with active transcription [76]. There are three major subtypes of H3K36 methyltransferases that have been identified: ASH1L, NSD1-3, and SETD2 [77]. All of them exhibit highly specific activities toward H3K36 in the context of nucleosomes [77].

A common feature of the H3K36 methyltransferase is a conserved autoinhibitory mechanism. In this mechanism an autoinhibitory loop between the SET and the post-SET motif blocks access of the histone tail substrate to the active site [78–80]. A similar autoregulatory loop is discussed above for the H3K9 methyltransferases Clr4 and potentially SUV39H1/2 [22,68]. However, H3K9 and H3K36 methyltransferases seem to utilise distinct mechanisms to reorient the autoinhibitory loop for substrate accommodation.

ASH1L catalyses the mono- and di- methylation of H3K36. It is essential for the maintenance of normal H3K36me2 levels at developmental genes [78,81]. ASH1L antagonises H3K27me3-mediated gene silencing by PRC2 in a molecular mechanism that is evolutionarily conserved from fly to human [82–85]. ASH1L exhibits low HMTase activity on its own, likely due to the inaccessibility of the substrate-binding site [78]. Ash1 is the *Drosophila* ortholog of human ASH1L. Ash1 associates with two other components, Mrg15 and Nurf55, to form the Ash1–Mrg15–Nurf55 complex [86,87]. Mrg15, but not Nurf55, stimulates the HMTase activity of Ash1 *in vitro* [86,87]. Accordingly, the depletion of Mrg15 leads to a significant reduction in H3K36me2 at Ash1 target loci *in vivo* [87].

Affinity purifications and subsequent mass spectrometry (AP-MS) and Co-IP revealed similar ASH1L-associating proteins in human cells [87]. The human ASH1L-associating proteins include MRG15, MRGX, RBBP4, and RBBP7 [87]. MRG15 and MRGX are two MRG domain-containing proteins, homologues of the fly Mrg15. RBBP4 and RBBP7 are two WD40-containing proteins, homologous to the fly Nurf55 [87]. As in the fly, human MRG15 can stimulate ASH1L enzymatic activity, with the MRG domain being the primary contributor [86,88,89]. These findings indicate that ASH1L resides within a complex that is evolutionarily conserved from fly to human. The ASH1L-containing complex is required for high H3K36 HMTase activity, and relies on subunit-induced allosteric activation to relieve the auto-inhibited state of the SET domain.

**Figure 4.**
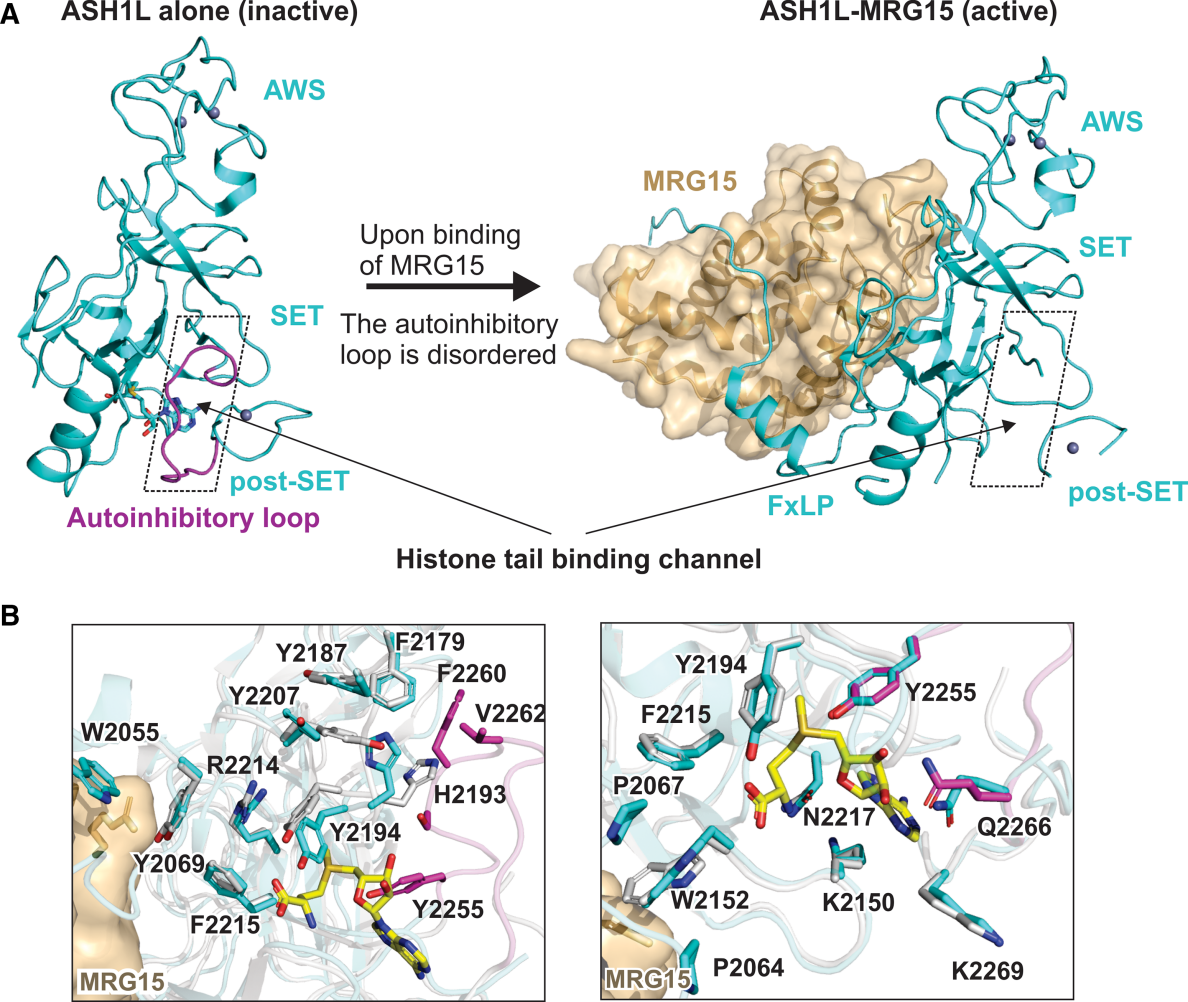
Subunit-induced conformational change stimulates the H3K36 methyltransferase ASH1L. (**A**) MRG15 binding releases the autoinhibitory loop of ASH1L. The autoinhibitory loop (in magenta) is ordered and blocks the histone tail-binding channel when ASH1L is not bound to MRG15 (left, PDB: 3OPE [78]). In the presence of MRG15, the autoinhibitory loop of ASH1L becomes disordered (right, PDB: 6AGO [88]). (**B**) Molecular mechanism for the displacement of the autoinhibitory loop in ASH1L. The superposition of ASH1L-MRG15 structure (structure in cyan, Left: 6AGO [88]; right: 6INE [89]) with ASH1L alone (grey, PDB: 3OPE [78]) indicates the binding of MRG15 induces a series of subtle conformational changes to destabilise the autoinhibitory loop. The autoinhibitory loop is ordered in the structure of ASH1L alone and is shown in magenta. The key residues involved in the displacement of the autoinhibitory loop are shown in sticks. In the left panel, Lee et al. [88] suggested that the hydrophobic network between MRG15 and ASH1L results in the reorientation of H2193 and Y2207, leading to the disorder of the autoinhibitory loop. In the right panel, Hou et al. [89] proposed that binding of MRG15 stabilises the conformations of P2067 and P2064, leading to the conformational changes of W2152, and further triggers a cascade of subtle rearrangements of the residues surrounding the SAM binding pocket. This results in a small shift of SAM and eventually leads to the disorder of the autoinhibitory loop.

Crystal structures of truncated ASH1L in complex with the MRG domain of MRG15 revealed how MRG15-induced allosteric stimulation of ASHL takes place [88,89]: a conserved FxLP motif in ASH1L binds to the MRG domain of MRG15. Upon binding of the MRG domain to ASH1L, the autoinhibitory loop of ASH1L becomes disordered without additional major conformational rearrangement of the SET domain. Following the release of the autoinhibitory loop from the lysine-substrate binding site, the substrate can now bind to the catalytic site and HMTase activity is allowed (Figure 4A). Lee et al. [88] suggested that two key residues, ASH1L H2193 and Y2207, undergo a conformational change upon MRG15 binding, leading to the displacement of the autoinhibitory loop (Figure 4B, left). Hou et al. suggested that two proline residues adjacent to the FxLP motif of ASH1L, P2064 and P2067, lead to subtle conformational changes surrounding the SAM binding site. As a result of these minor conformational changes the autoinhibitory loop becomes disordered and the enzyme is active (Figure 4B, right) [89].

Subunit-induced structural rearrangement of ASH1L is the first case of allosteric regulation identified in H3K36 methyltransferases [77]. Evidence implies that allosteric regulation might also take place within the NSD family and SETD2 H3K36 methyltransferases. Specifically, a Cryo-EM structure of the fungal Set2 bound to a nucleosome revealed direct interactions between the Set2 to the nucleosomal DNA [90]. These Set2-DNA interactions induce conformational change of the autoinhibitory loop and allows the access of the histone tail [90]. DNA was also proposed to serve as an allosteric effector of NSD2 [91]. The NSD family exhibits higher enzymatic activity and H3K36 selectivity with nucleosome substrate compared with the histone substrates [79,91,92]. Consistent with these observations, NSD2 and NSD3 undergo conformational changes in the autoinhibitory loop region upon nucleosome binding [93,94]. In the absence of nucleosome binding, the autoinhibitory loop is stabilised in the active site of NSD2 or NSD3 through inhibitory interactions with the SET domain [93,94]. Upon nucleosome binding, the SET domain interacts with the histones and the nucleosomal DNA to replace the interaction with the autoinhibitory loop [93,94]. Consequently, the autoinhibitory loop is pushed away from the active site, making the substrate-binding site accessible. It is still to be determined whether displacing the autoinhibitory loop upon nucleosome binding is a conserved mechanism within other members of H3K36 methyltransferases.

Collectively, two mechanisms for the allosteric activation were proposed to relieve the autoinhibitory state of H3K36 methyltransferase: (1) subunit-induced allosteric regulation and (2) nucleosome binding-induced conformational change of the autoinhibitory loop. Both mechanisms might safeguard H3K36 methyltransferases by preventing their activity in the absence of an assembled complex or away from chromatin.

## Subunit-induced conformational changes in H3K4 methyltransferases adjust their enzymatic activity

The methylation of H3K4 is generally recognised as a mark of gene activation [95–98]. H3K4 methylation is catalysed by the SET1/mixed lineage leukemia (MLL, also term KMT2) family. All these H3K4 methyltransferases share a SET domain that is evolutionarily conserved throughout eukaryotes [96–98]. Set1 is the sole H3K4 methyltransferase in yeast and there are three subgroups of MLL proteins in Drosophila: Trx, Trr and Set1. With gene duplication during mammalian evolution, the homologous MLL family in human further expanded to six members that are functionally distinct: the Trx-related MLL1 and MLL2, Trr-related MLL3 and MLL4, and Set1-related SETD1A and SETD1B [96–98]. Given the essential role of the MLL family in gene regulation, dysregulation of MLL family proteins is linked to different human diseases, such as acute myeloid and lymphoid leukemia and Kabuki syndrome [99–102].

**Figure 5.**
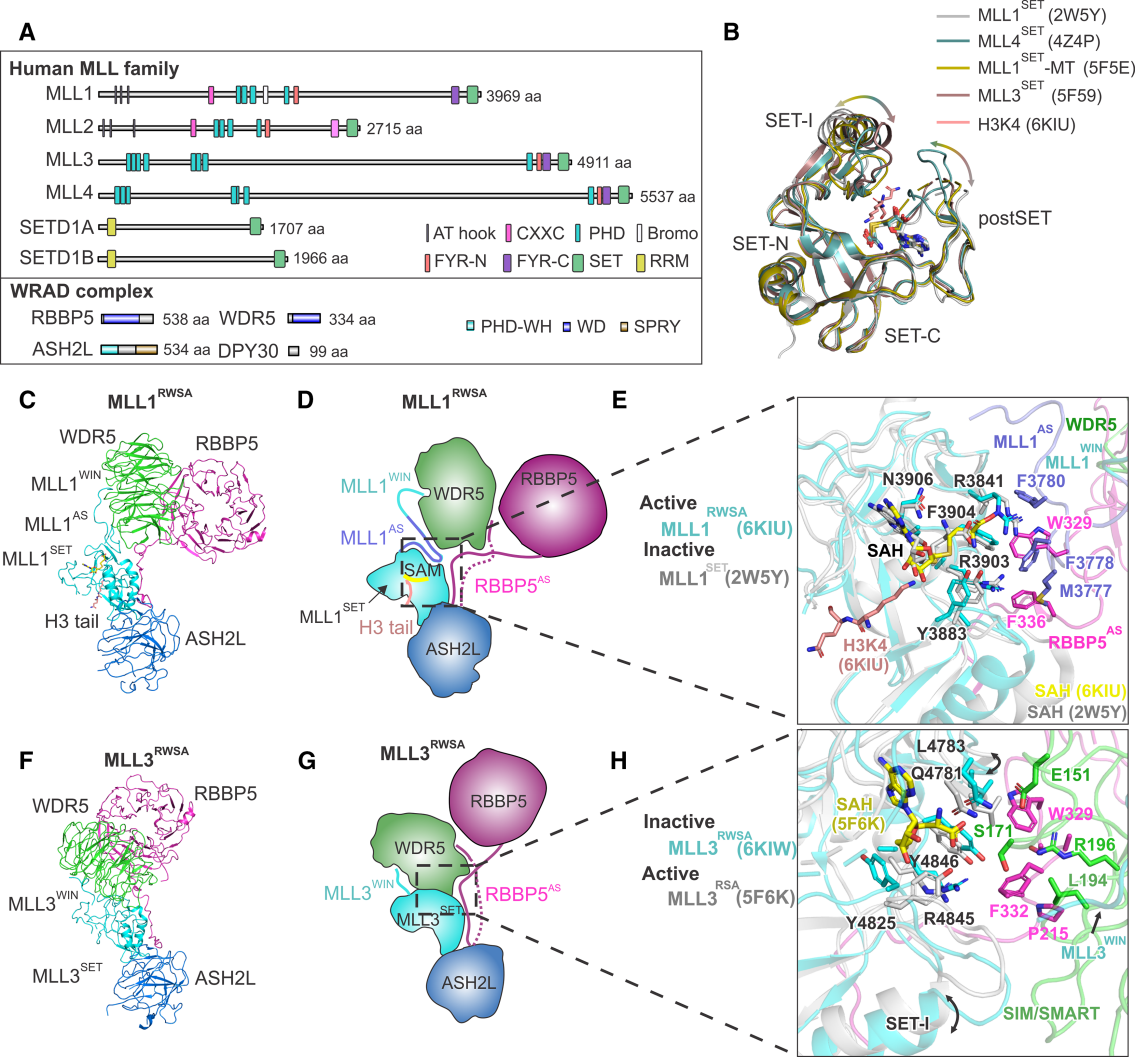
Subunit-induced conformational change stimulates the MLL family H3K4 methyltransferases. (**A**) Domain organisation of the human MLL family proteins and subunits of their cofactor WRAD complex. (**B**) Superposition of SET domains of different MLL family proteins (protein names and PDB accessions are indicated in the figure). The different conformations of the SET-I and post-SET regions as captured among different MLL proteins are highlighted by arrows. (**C**) Cryo-EM structure of the human MLL1-WRAD complex (PDB 6KIU [109]; determined in the presence of a nucleosome containing monoubiquitin on H2BK120, which was omitted for this representation). MLL1^RWSA^ stands for RBBP5–WDR5–MLL1^SET^–ASH2L complex. MLL1^AS^ is the activation segment of MLL1. RBBP5^AS^ is the activation segment of RBBP5. MLL1^WIN^ is the WDR5 interaction motif within MLL1. Protein subunits are shown in different colours. (**D**) Schematic representation of the human MLL1 complex as in (**C**), using the same colour code. (**E**) Structural comparison of the SET domain as determined for MLL1 alone (PDB: 2W5Y [23]) or in a complex with the cofactor WRAD (pdb: 6KIU [109]). The key residues mediating interactions between MLL1 and RBBP5 are shown in sticks. Subunits and chains are colour coded as indicated in the figure. (**F**) Cryo-EM structure of the human MLL3–WRAD complex, as determined together with a nucleosome containing monoubiquitin on H2BK120 (PDB: 6KIW [109]; the nucleosome is not shown). MLL3^RWSA^ stands for the RBBP5–WDR5–MLL3^SET^–ASH2L complex. RBBP5^AS^ stands for RBBP5 activation segment. MLL3^WIN^ stands for WDR5 interaction motif. Protein subunits are shown in different colours. (**G**) Schematic representation of the human MLL3 complex as in (**F**), using the same colour code as above. (**H**) Structural comparison of the MLL3 SET domain as determined in the absence (PDB: 5F6K [105]) or presence (PDB: 6KIW [109]) of the WRAD cofactors. The key residues that mediate interactions between MLL3, RBBP5 and WDR5 are shown in sticks. Subunits and chains are colour coded as indicated in the figure.

All six members of the MLL family in humans are large multi-domain proteins, with the highly conserved catalytic SET domain in their C-terminal region. Contrarily, the highly divergent N-terminal regions of MLLs function in their recruitment and modulating their enzymatic activity [95,103] (Figure 5A). A common feature of MLL from yeast to humans is that additional subunits are required to stimulate the enzymatic activity and the methylation level of their product [103,104]. In yeast, the Set1 protein on its own exhibits a low methyltransferase activity. Full catalytic activity of Set1 is achieved once it is associated with Cps30 (Swd3), Cps50 (Swd1), Cps60 (Bre2) and Cps25 (Sdc1) to form a stable Set1 complex, also known as COMPASS (complex of proteins associated with Set1) [96]. The mammalian ortholog of the yeast Set1 complex includes WDR5, RBBP5, ASH2L and DPY30, and is collectively referred to as the WRAD complex. The WRAD complex forms a stoichiometric complex with MLL family proteins through direct interaction with their catalytic C-terminal SET domain [95,96].

Crystal structures of the isolated SET domain from MLL1, MLL3 and MLL4, in a complex with S-adenosylhomocysteine (SAH), revealed either an open or a closed conformation [23,103,105] (Figure 5B). A closed conformation of the cofactor- and substrate-binding channel is thought to favour substrate binding and methyl transfer [23]. Molecular dynamics simulations revealed the high dynamics of the SET-I region [105], which forms the substrate and cofactor binding site together with the post-SET region (Figure 5B). This analysis supports a model where WRAD binding reduces the flexibility of SET-I and post-SET by inducing a conformational change of the SET-I region (Figure 5C,D). Interactions attributed to WRAD binding thus stabilise the catalytic domain of MLL proteins in its fully activated conformation [105]. A model where WRAD binding is required to stabilise the substrate-binding site is further supported by structures of the yeast COMPASS and the human MLL1-WRAD complexes [106–110].

The activation segments in RBBP5 and MLL1, referred to as RBBP5^AS^ and MLL1^AS^, respectively, are loop regions critical for the activation of MLL1 [109]. Both loops form extensive hydrophobic interactions with the SET domain of MLL1 (MLL1^SET^). Consequently, the SET-I region is locked into a closed active conformation with respect to its inactive apo-form (Figure 5E) [23,109]. In the active conformation of MLL1, SAH is shifted away from the substrate lysine-binding site, leaving more room to accommodate the lysine substrate. Overall, this structural rearrangement leads to a higher degree of methylation [109].

A loop within the WD40 domain of Cps30/Swd3 (the yeast ortholog of WDR5) modulates the activity of the SET domain of the yeast Set1 by contacting the SET-I region. This regulatory loop is referred to as SIM (SET-I interacting motif) or SMART (Set1 Methyltransferase Activity Regulator) motif [107,108]. However, similar interactions were not observed in the human MLL1-WRAD complex owing to different interactions centred by MLL1^AS^ [109]. Instead, the human WDR5 is pushed away from MLL1^SET^ and no direct interaction occurs between them [109]. The SET domains within all six members of human MLL are well conserved, with the exception of the MLL^AS^ [109]. It suggests that this variability of MLL^AS^ may generate different interfaces between RBBP5, WDR5 and MLL^SET^. These variations between MLLs may lead to the induction of different conformational changes to their SET domains to vary their enzymatic activities [109].

WDR5 was shown to inhibit the activity of MLL3 for histone H3 peptides [111] and nucleosome substrates [109]. When MLL3^SET^ is associated with WRAD, it only displays weak mono-methyltransferase activity on nucleosomes [109]. The key hydrophobic residues of MLL1^AS^ are absent from MLL3, leading to a different RBBP5–WDR5–MLL3 interface compared with MLL1 (Figure 5F,G) [109]. Unique to MLL3 are the direct interactions between the WDR5 SIM/SMART loop [107,108] to the SET-I motif of MLL3 (Figure 5H) [109]. These interactions induce a slightly opened conformation in the active site of the MLL3 [109], in accordance with reduced activity. Accordingly, MLL3 has been recognised as a monomethyltransferase of H3K4 *in vivo* [112].

H2BK120ub stimulates the enzymatic activity of the MLL family, but the mechanism appears different within the 6 members of MLL family [113]. In both yeast COMPASS and the human MLL1-WRAD, H2BK120ub has little to no effect on nucleosome binding [114,109]. An arginine-rich motif (ARM) was identified in the yeast Set1 and was shown to be critical for the H2BK120ub-dependent stimulation of the COMPASS [114,115]. H2BK120ub was proposed to allosterically activate yeast Set1 through stabilising its ARM region onto the acidic patch of the nucleosome. The stabilisation of the Set1 ARM induces minor structural changes within the catalytic domain of Set1 [115].

Interestingly, the ARM region is only conserved in the Set1 human orthologs SETD1A and SETD1B, but not in the human MLL1-4, suggesting H2BK120ub activates MLL1-4 using a different mechanism. Furthermore, cryo-EM structures of human MLL1 and MLL3 with a nucleosome-containing mono-ubiquitinated H2BK120 did not detect direct interactions between the ubiquitin and the SET domain [109]. Further studies are required to elucidate how different MLL-family proteins are activated by the H2Bub mark. Such mechanistic studies will be valuable to determining the functional consequences of H2Bub-mediated regulation of H3K4 methyltransferases *in vivo* [116]

Variations between WRAD-MLL interfaces induce different conformational changes within the SET domains of different MLLs. These variations account for the different enzymatic activities and methyl-lysine levels of MLL1 compared with MLL3. At this time, there are no structures of SETD1A or SETD1B with their nucleosome substrates. Future structural studies are still required to reveal how the WRAD complex regulates these two human homologues of the fly Set1 H3K4 methyltransferase. But overall, MLL family enzymes need to associate with other protein subunits to be active, similar to the mechanisms described above for the regulation of EZH2 and ASH1L.

## Allosteric effectors of HKMTs: a path for selective epigenetic drugs

Epigenetic drugs designed to target chromatin-modifying enzymes are considered promising therapeutics, with some already approved for clinical usage [117,19]. Histone lysine methyltransferases are considered sought-after targets for drug development, given their involvement in various diseases. The majority of small-molecule inhibitors of HKMTs are substrate competitors, designed to compete with SAM or histone substrates. Yet, SAM-competitive inhibitors have to be sufficiently polar to exploit the SAM binding site and hydrophobic enough to penetrate the cell membrane, which has proven to be challenging [118]. A number of SAM-competitive inhibitors of EZH2 had limited therapeutic potential, given their short half-life and low permeability in cell-based assays [44]. Another challenge with developing selective competitive inhibitors is the high resemblance between SET domain proteins of the same family, especially in the lysine binding channel. Indeed, concerns for cross-reactivity between epigenetic drugs and off target adverse effects have already been flagged [19].

Contrarily to substrate-competing inhibitors, allosteric inhibition is a promising strategy to target specific histone lysine methyltransferases in a specific context. The allosteric regulation of histone lysine methyltransferases is triggered by diverse effectors and mechanisms, and commonly takes place in a context-dependent manner. These diverse molecular mechanisms open a path for the development of highly selective drugs to target HKMTs in specific disease contexts [19,118]. Additionally, targeting allosteric sites provides an alternative treatment against tumours already resistant to catalytic inhibitors [119]. Indeed, a number of small molecules targeting the allosteric sites of the SET-domain containing HKMTs were developed, some of which are already under clinical trials.

While targeting non-catalytic sites in HKMTs in order to perturb their allosteric regulation is an emerging theme, a good amount of work was already done in this space on PRC2. Small molecules that were designed to bind the allosteric site of the PRC2 regulatory subunit EED disrupt its interaction with the H3K27me3 mark. Some of these EED inhibitors are highly effective in inhibiting the histone methyltransferase activity of PRC2 [120–123]. Among them, EED226 induces a conformational change upon binding to EED and these interactions lead to the loss of PRC2 activity, with a similar cellular effect as the SAM-competitive inhibitors targeting the active site [120]. Importantly, EED226 exhibits inhibitory activity toward PRC2 containing a mutant EZH2 protein resistant to SAM-competitive inhibitors [120]. Hence, EED226 demonstrates the potential in targeting allosteric sites of KHMTs in cases where mutations in the active site prevents the usage of competitive inhibitors. MAK683 is a small molecule from Novartis, designed from the scaffold of EED226 and is currently under phase I/II clinical trials for the treatment of lymphoma^[1]^ [19,124].

Another strategy of targeting PRC2 is to block protein–protein interactions (PPI) between the catalytic subunit EZH2 and the regulatory subunit EED. This was done by a stabilised α-helix peptide, designed to mimic an EED-interacting helix from EZH2 to disrupt its interactions with EED. Accordingly, the peptide destabilises PRC2 and selectively inhibits PRC2 in cancer cells [125]. Following this strategy, the small molecules astemizole [126] and wedelolactone [127] were developed to destabilise the interactions between EED and EZH2. EED-degron small-molecules are another complementary strategy for targeting the regulatory subunit EED. The proteolysis targeting chimeras (PROTACs) bind to EED and promote ternary complex formation with an E3 ubiquitin ligase. Although EED hosts the binding site for allosteric effectors, not the catalytic center, targeting EED by degron molecule typically leads to the rapid degradation of both EED and the catalytic subunit EZH2 [128,129].

Further studies to reveal how allosteric regulation of SET-domain histone lysine methyltransferases occurs will expand the panel of druggable epigenetic modifiers in disease.

## Concluding remarks

More than 100 lysine methyltransferases have been reported in humans [18]. Allosteric regulation is common in enzymes and it also plays a role in histone-modifying complexes [117]. We discussed in detail two major mechanisms for allosteric regulation of HKMTs: (1) subunit-induced conformational change and (2) external stimulus by a nucleosome or a specific histone mark, as in the read-write mechanism. Subunit-induced regulation could safeguard HKMTs from becoming active externally to the context of their complex. The second category of external stimuli is rather broad, and allows HKMTs to sense their environment and tune their enzymatic activity accordingly. Further characterisation of HKMTs is required to reveal the full cohort of their effectors. These might include additional histone marks and potentially also DNA and RNA.

At this point in time, some HKMTs are incompletely characterised, including their substrate specificity and their regulation of catalysis [130]. Advances in cryo-EM techniques may allow better understanding of how allosteric regulation of histone lysine methyltransferases takes place, mechanistically. Structures of HKMTs in complexes with their substrates and effectors would likely allow for their different active states to be captured in action. The rapidly developing artificial intelligence networks used to predict 3D structure [131,132] may also accelerate the discovery of more allosteric sites and hold great promise for drug discovery. Furthermore, chemically synthesised-designer chromatin provides a powerful tool to quantitatively determine the methyltransferase kinetics under different contexts [75,133]. Collectively, these will likely reveal the roles of various effectors in the modulation of histone lysine methyltransferase.

## Perspectives

- Histone lysine methyltransferases (HKMT) are commonly subjected to allosteric regulation, allowing for their regulation in a context-specific manner.
- Advances in structural biology allow revealing how allosteric regulation of some HKMTs takes place at the molecular level. Future structural studies of intact HKMTs complexes together with their chromatin substrates *in vitro* and *in situ* will likely reveal the full variety of mechanisms for their allosteric regulation.
- The consequences of the allosteric regulation of HKMTs in development and disease remains a key knowledge gap. Advances in genome modification techniques and structural biology will likely allow this knowledge gap to be closed, and open a path to targeting allosteric sites and effectors of HKMTs in disease.

## Note

[1] ClinicalTrials.gov Identifier: NCT02900651

## Open Access

Open access for this article was enabled by the participation of Monash University in an all-inclusive *Read & Publish* pilot with Portland Press and the Biochemical Society under a transformative agreement with CAUL.

## Abbreviations

AP-MS: Affinity purifications and subsequent mass spectrometry
ARL: autoregulatory loop
ARM: arginine-rich motif
CD: chromodomain
COMPASS: complex of proteins associated with Set1
HKMTs: Histone lysine methyltransferases
MLL: mixed lineage leukemia
PcG: polycomb-group
PPI: protein–protein interaction
PRCs: Polycomb repressive complexes
PROTACs: proteolysis targeting chimeras
SAH: S-adenosylhomocysteine
SAM: S-adenosyl-L-methionine
SET Su(var)3-9: Enhancer of Zeste and Trithorax
SET-I: SET insert
SIM: SET-I interacting motif
SMART: Set1 Methyltransferase Activity Regulator motif
SRM: stimulatory-responsive motif
WRAD complex: WDR5, RBBP5, ASH2L and DPY30
